# “A Labour of Love”: Active Lifestyle Entrepreneurship (Occupational Devotion) During a Time of COVID-19

**DOI:** 10.3389/fspor.2021.624457

**Published:** 2021-04-22

**Authors:** Richard Keith Wright, Cindy Wiersma, Richard Opara Ajiee

**Affiliations:** ^1^Sports Performance Research Institute New Zealand, Auckland University of Technology, Auckland, Auckland, New Zealand; ^2^School of Hospitality and Tourism, Auckland University of Technology, Auckland, New Zealand

**Keywords:** occupational devotion, active lifestyle entrepreneurship, creative analytical practice, COVID-19, personal narrative, serious leisure

## Abstract

The Novel Coronavirus (COVID-19) crisis management strategies adopted by world leaders across the globe in 2020 impacted the work-life balance of billions of people. Entire populations were forced to stay at home and maintain a safe distance from family members, friends, colleagues, and customers. Occupational devotion is defined as a feeling of strong, positive attachment to a form of self-enhancing employment, where achievement and fulfillment are high, and the core activity has such intense appeal that the line between this work and leisure is virtually erased. Although it is not a new concept, this area of the serious leisure perspective has been largely overlooked by scholars observing the world of sport events and entrepreneurship. Using Creative Analytical Practice (CAP), a post-qualitative methodology, we present the personal narrative of a New Zealand-based active lifestyle entrepreneur who, as a result of a nationwide COVID19 lockdown, was forced to re-assess his long-established occupational devotion. Our co-constructed story offers an emotive insight into the personal cost and consequences of finding yourself living in a lockdown.

## Episode One: A Team of Five Million

“The coronavirus COVID-19 pandemic is the defining global health crisis of our time and the greatest challenge we have faced since World War Two… But the pandemic is much more than a health crisis, it's also an unprecedent socio-economic crisis. Stressing every one of the countries it touches, it has the potential to create devastating social, economic and political effects that will leave deep and longstanding scars… The International Labor Organization estimates that 400 million jobs could be lost. Every country needs to act immediately to prepare, respond, and recover” (United Nations Development Programme, 2020).

In March 2020, the International Monetary Fund's (IMF's) chief economist referred to the highly infectious respiratory disease as “a crisis like no other” (Chan, 2020). The Novel Coronavirus (COVID-19) crisis management strategies adopted by world leaders across the globe has directly impacted the work-life balance of billions of people with millions forced to stay at home and maintain a safe distance from family members, friends, colleagues and customers. The social lockdowns and strict restrictions to human movement, witnessed on all seven continents, ruthlessly exposed the socially-constructed boundaries that separate what is and what is not an essential part of twenty-first century society. In an attempt to save lives and reduce the spread of COVID-19, all large social gatherings were canceled or postponed, including some of the world's most iconic sports events. There was no Wimbledon (for the first time since 1945). There was no Monaco Formula One Grand Prix (for the first time since 1954). Likewise, the British (Golf) Open, the European Football Championships, and the Games of the XXXII Olympiad (aka Tokyo 2020) were all unceremoniously removed from the 2020 sporting calendar.

New Zealand's Director-General of Health, Dr Ashley Bloomfield, publicly reported the country's first case of COVID-19 in an announcement made on Friday February 28th, 2020. The following week, Prime Minister Jacinda Ardern revealed her government's COVID19 crisis management strategy. The goal was to go hard, act fast and completely eliminate/eradicate all communal transmission as quickly as possible. It was a strategy based on the recommendations of scientists from within the field of public health and epidemiology. For the aggressive strategy to work, the government needed everyone to follow the new rules of social engagement.

Four COVID-19 Alert Levels (C-19 AL) were presented to the public via a nationwide address on March 21st March. They were labeled; Prepare (Level 1), Reduce (Level 2), Restrict (Level 3) and Lockdown (Level 4). Each CL-19 AL came with their own range of strict conditions and control measures and the nation was immediately placed into CL-19 AL3. This was changed to C-19 AL4 2 days later, as the Prime Minister declared a State of National Emergency starting at 12:21 p.m. on 25 March 2020. The nationwide lockdown lasted for 49 days. All social gatherings were outlawed, with only essential workers allowed to leave their place of residence (referred to as “a bubble”). The only businesses that were allowed to remain open were those deemed “essential” services (e.g., supermarkets, pharmacies, petrol stations etc.). Gyms and sports fields/facilities were closed and all events were either canceled or postponed. The New Zealand border was closed to all foreign nationals and those who were eligible to enter on “mercy” or “repatriation” flights were required to self-isolate for 2 weeks. Light/low intensity physical activity such as walking, jogging or cycling within one's local neighborhood was permitted, but only with the members of your bubble.

Whilst some non-essential businesses were allowed to reopen in late April, as the country moved into C-19 AL3, it wasn't until the nation moved into CP-19 AL2 on the 13th of May that schools and other childcare facilities were allowed to reopen. All bars and restaurants were also allowed to reopen at this point, but under strict conditions that limited numbers and enforced social distancing. New Zealand entered C-19 AL1 at 11:59 p.m. on Monday 8 June, 2020, following 2 weeks of no community cases being recorded. No sport fixtures or events were permitted until New Zealand had been in C-19 AL1 for a couple of weeks. By the end of June, life in New Zealand had essentially returned to “normal.” Events hosting over 100 people were once again permitted and the domestic winter sports leagues, both amateur and professional, were able to commence.

Our paper shares the personal narrative of an active lifestyle entrepreneur living in New Zealand. Having set the scene within the opening paragraphs, the rest of the paper has been split up onto a trilogy of overlapping episodes. Episode two introduces the concepts of active lifestyle entrepreneurship and occupational devotion. Episode three is split into two halves. The first focuses on the philosophies, principles and practices of post-qualitative narrative inquiry, more specifically the employment of Creative Analytical Practice (CAP). The second presents a personal narrative entitled “*THE* event of our lifetime (got to run).” Finally, the fourth episode offers our socially constructed interpretations of the method(s) employed and the meaning(s) extracted. Our aim is to establish a meaningful and memorable connection between the reader and the subject of an evocative personal narrative.

## Episode Two; Active Lifestyle Entrepreneurship

The globalization, commercialization and commodification of sport has led to a noticeable increase in the number of entrepreneurs looking to establish their own space in a heavily congested and highly competitive active economy (Jones et al., 2020; Ratten, 2020). According to Finch et al. (2019, p.4), an “active economy” “incorporates all organizations who participate in, or contribute to, improving individual and/or community level well-being through the development and delivery of sport, physical activity and active recreation experiences”. They identify ten interdependent sectors that co-exist within an active economic ecosystem, including organized sport and active recreation (Finch et al., 2019). Organized sport is characterized as structured and competitive physical exertion requiring skills and rules, whereas active recreation incorporates more relaxed, sociable leisure experiences primarily encountered for health and well-being, as well as entertainment and enjoyment.

In 2019, Scott Jones, the creator of a social enterprise called “Athletes On Fire,” defined the active entrepreneur as “someone who pursues an entrepreneurial endeavor in a field that inspires, teaches, motivates, or trains people to move, or, an entrepreneur that lives an active and adventurous lifestyle while maintaining an entrepreneurial endeavor” (Jones, 2019). Paula Gregorowicz, founder of “The Paula G Company, differentiates the active entrepreneur from the athletic entrepreneur, placing a greater emphasis on an individual's passion for “something… anything that makes your heart sing”, whether it be gardening, singing, cooking or running” (Gregorowicz, 2015). Similarly, the term “lifestyle entrepreneurship” was first coined to describe local artisan or craft-based business owners looking to establish a better work-life balance (Bredvold and Skalen, 2016).

Burns (2001) defines lifestyle enterprises as businesses that not only provide adequate income, but also allows the owner-manager to engage in an area of activity that brings them personal pleasure. A lifestyle entrepreneur is therefore someone who creates a new business venture that aligns with their personal values, interests and identities (Wright and Wiersma, 2019; Jones et al., 2020). According to Shaw and Williams (2004), lifestyle entrepreneurs are typically older male adults who enjoy being their own boss. Furthermore, research suggests that many have retired or resigned from former better paid professions, and/or moved to a new areas with the intention of generating enough income to sustain their lifestyle (Peters et al., 2009). They are typically experienced consumers (hobbyists) and product innovators who share a number of personal characteristics, including a strong desire to enhance their quality of life and that of their family (Peters et al., 2009). Their goal isn't to establish a business that they can grow and/or harvest, but one that will be low maintenance and enjoyable to own/operate.

Marchant and Mottiar (2011) divided the lifestyle entrepreneurship market into those “constrained” and those “non-constrained.” Drawing on occupational socialization theory, Kim and Longest (2014) determined that there was a direct correlation between an entrepreneur's past professional background and the number of people they were willing to involve in their new business idea. Those coming from venture-specific profit-driven industries were found to be more likely to opt for solo ventures or small collaborations, whilst those coming from more interactive people-driven occupations were more likely to seek and recruit a number of new business partners. Although the success and sustainability of new ventures are typically assessed by the speed in which they are progressing, if not growing, Kim et al.'s (2015) study into the viability of American leisure-based entrepreneurship found that social lifestyle entrepreneurs were content to play the long game. Lifestyle entrepreneurs situated in New Zealand's tourism sector, for example, were found to specifically market their product toward consumers who shared their socio-cultural values (Ateljevic and Doorne, 2000).

### Occupational Devotion: the Sleeping Giant of the Serious Leisure Perspective

Serious leisure is defined as the “systematic pursuit of an amateur, hobbyist or volunteer activity sufficiently substantial and interesting in nature” (Stebbins, 1992, p.3).

The consumers of serious leisure typically navigate their way through four or five overlapping “career” phases (Stebbins, 2014). The resources invested in the beginning, development, establishment, maintenance and decline stages can vary, however, with one's progression being driven by a range of social, cultural, and historical factors (Stebbins, 2014). Serious leisure consumers have also been found to share a number of personal traits, many of which are also shared by active lifestyle entrepreneurs (Jones et al., 2020). These including the drive to persevere, the desire to pursue a career, the willingness to put in effort in order to gain skill and knowledge, the ability to realize the special benefits attached to self-fulfillment, self-expression and group accomplishment, the ability to create/cultivate of an attractive personal and social identity and, lastly, the ability to maintain a unique ethos through active involvement within your social world (e.g., an internally recognizable group of likeminded people) (Stebbins, 2014a).

Although the concept of serious leisure has been explored on numerous occasions over the last 30 years, surprisingly little attention has been paid to the subset of “devotee work,” defined as “an activity in which participants feel a powerful devotion… to an occupation that they are proud to be in” (Stebbins, 2014a, p, 4). Stebbins (2014b) justifies the inclusion of occupational devotion within the broader serious leisure perspective (SLP), arguing that those who experience such an intense positive attachment and a sense of high-level achievement (on a day-to-day basis) can effectively remove the boundary between work and leisure. Occupational devotion is defined as a feeling of strong, positive attachment to a form of self-enhancing employment where the core activity has such intense appeal that the line between this work and leisure is virtually erased (Stebbins, 2004).

Stebbins (2004) split the work devotees from the non-devotees, using the following seven criteria; skill, knowledge/experience, variety, creativity/innovativeness, control, aptitude/taste, social/physical milieu and the ability to work in a team. In terms of personal characteristics, the occupational devotee was identified as someone with the following five strongly seated socio-cultural values; success, achievement, freedom of action, individual personality and activity (being involved in something) (Stebbins, 2009). Their devotion is evident in their everyday actions, lifestyle, motivation, and social relations. More so, it helps to define their personal and social identities (i.e., who they are and how they are seen by others) (Stebbins, 2009). According to Stebbins (2009), occupational devotees acquire a combination of skills, knowledge and experiences that offers ample opportunity for creativity or innovation. The concepts of occupational devotion and devotee work were framed within the four broad social contexts of history, religion, work, and leisure (Stebbins, 2004). Gender, social class, and social character have also been used to help researchers explore the antecedents of occupational devotion (Stebbins, 2004).

On the subject of remuneration, Stebbins (2004) proposes that there are three critical orientations present within the concept of occupational devotion. The first exists when an work devotee is primarily seeking payment that allows them to continue pursuing the core activity (i.e., they seek to be paid so that they may continue working). Stebbins (2004) suggests that the true value for those who fall within this cohort comes from being in a position to routinely carry out their fulfilling work. Money is not the supreme value to principles-led devotees, who search for little more than the minimum needed to sustain their preferred lifestyle. The second orientation is that of acquisition. This covers the work devotees who seek an occupation that allows them to live comfortably as opposed to passably (i.e., they seek extrinsic rewards and future wealth and are happy to relocate or reinvent themselves to get it). The third group represent a combination of the first two orientations. Those deemed to adhere to “Principled-acquisitive orientation” seek to be paid so they may continue working in an role that gives them pleasure, but see no reason why they cannot sooner or later generate enough profit to sustain a comfortable living (i.e., they seek a healthy but profitable work-life balance).

Although occupational devotees are not immune to feelings of work-based frustration, anger and (self)doubt, the strong emotional connections felt toward the thing they do for a living (their livelihood) ensures that the positive aspects soon negate any such negativity (Stebbins, 2009). Four somewhat broad and non-exclusive types of devotee work are identified within under the banner of occupational devotee within the SLP framework, these being; liberal professions, consulting/counseling occupations, some skilled trades, some small businesses (Stebbins, 2014a). Stebbins acknowledged the difficulty in studying occupational devotion from an entrepreneurial perspective from the outset, blaming the scarcity of data available on small business entrepreneurs (Stebbins, 2004). He also noted the lack of research in which occupational devotees have claimed to have achieved something important or that they have been successful in life, concluding that, “compared with others in their reference groups, they [occupational devotees] have developed considerable knowledge and skill and acquired considerable experience, all of which they have applied with a certain level of creativity or innovation. But many devotees, unlike some other kinds of workers, cannot measure their achievement and success in remunerative terms for high pay by no means always flows from occupational devotion” (2004, p. 18).

Durieux and Stebbins (2010) suggest that, “to find serious leisure through social entrepreneurship, you must have a sense of commitment and a moral obligation to your enterprise and its target of benefits. These two attributes are key in motivating you to serve as a volunteer in your own social enterprise… Commitment and leisure may sound like an odd couple, but in serious leisure, commitment is a central condition” (pp. 47-48). In Hjorth (2007) proposed that those who study entrepreneurship needed to broaden their resource-based focus to include the time of opportunity creation as well-opportunity recognition, evaluation and exploitation. His recommendation was for the adoption of more humanistic narrative-based methods that allow researchers to look at entrepreneurship from personal perspective.

The aim of this episode was to introduce you to the concepts of active lifestyle entrepreneurship and occupational devotion, the latter of which remains an area of the serious leisure perspective worthy of further exploration, especially within the field of sport-based entrepreneurship. As noted within the introduction, the next episode is split into a couple of parts/halves. The first half provides an overview of the paradigms and principles behind post-qualitative narrative inquiry. The second half offers our attempt at employing creative analytical practice within a post-qualitative piece of narrative inquiry.

## Episode Three; Creative Analytical Practice (The Social Science of Storytelling)

Coffey (1999, p. 11) claims that the search for new knowledge is always “situated, partial, local, temporal and historically specific.” Narrative inquiry is the study of how human's construct and consume the world through the creation and subsequent sharing of stories (Bruner, 1990). Narrative accounting (or storytelling) allows a series of individual stories to be structured in a manner and direction that demonstrate a sense of movement through time and space (Gergen and Gergen, 1986; Polkinghorne, 1997). Put another way, narratives provides the map that can save people from getting lost in a sea of nostalgia (Ricoeur, 1991). A narrative can also shape our social identity and allow us to make sense of our present day priorities (Clandinin and Connelly, 2000). Narrative-based inquiry not only showcases, but also situates the complexity and messiness of the lived experience (Wright, 2017).

A narrative is broadly defined as “the ordering and connecting of particular subjects, events, actions, and experiences in a causally or temporally meaningful sequence or whole” (Wright and Blair, 2016, p.219). A narrative is not only an essential structure in human meaning-making, but also the discourse form which can express the diachronic perspective of human actions (Smith and Sparkes, 2009, Wright, 2017). It is widely considered to be the heart, the soul and the structure of any fictional or non-fictional story (Papathomas, 2016). Personal narratives are typically produced to inspire reflection and a period of critical self-examination (Richardson, 1999). They can appear as a single thread or as a collection of numerous threads (plots) that have been weaved together to form a multi-layered account a past lived experience (Polkinghorne, 1997). The process of extracting, removing and combining multiple memories into a single narrative is commonly referred to as emplotment (Polkinghorne, 1997). According to Papathomas (2016, p.39), narrative analysis represents “the cardinal approach to making sense of our worlds.”

Narrative researchers only plot the particular, as opposed to the general, seeking to showcase the inherent complexities that accompany individuality as opposed to summarizing the broader patterns that exist within a community (Bruner, 1990; Clandinin and Connelly, 2000). Creative Analytical Practice (CAP) is a form of contemporary ethnography that invites the reader into the research, allowing them to unearth new learnings from/through the consumption of personal narratives and other types of emotive, socially-constructed, conversational representations (Richardson, 1999; Parry and Johnson, 2007; Berbary, 2015; Wright, 2019). CAP ethnographers package and present stories of lived experiences, both their own and those of others (Richardson, 1999). These stories are typically shared in the form of evocative autoethnography or creative non-fiction, with the emphasis placed on the “believability” and “trustworthiness” of the content (as opposed to its generalisability) (Smith, 2016; Wright, 2019).

CAP “brings to the consciousness some of the complex political/ideological agendas hidden in our writing” (Richardson, 2000, p.254). CAP was born in the 1990s, inspired by a crisis of representation that spread across the humanities and social sciences and effectively challenged the way things had traditionally been done. It offers interpretivists, humanists and social constructionists a literary-inspired means of sharing personal stories, including their own (Richardson, 1999). CAP ethnographers are storytellers who relate to a host of critical “post-” paradigms, including poststructuralism, posthumanism and postmodernism (Berbary, 2015). Berbary (2015) suggests that CAP ethnographers can benefit from more unstructured guides used for more narrative-style semi-structured interviews. She also notes the possibilities attached to moving from specific stories to broader concepts, focusing on the combination of existing themes/ideas and the creation of poly-voiced, dialogic, juxtaposed narratives, composites, or visual forms. She concludes that CAP can be applied to post-qualitative studies that elicit thick, rich, contextualized stories, but also warns that it is “almost impossible to move in the opposite direction from broad information to in-depth stories” (p.37).

The epistemology behind CAP is based on the concept of nascency (Saldaña, 2005). In essence, the judgement of value and validity is not for the author to dictate, but for each individual audience member to determine in their own time (Saldaña, 2005). The goal of the storyteller is to temporarily transport his/her audience into a place that they are able to recognize, if not a place that they have previously visited (Wright, 2019). Wright (2019) adds that one of the benefits of employing CAP is its proven ability to provoke a reaction and strike a chord with a global audience, including those who would not normally consume academic discourse. The most appropriate assessment criteria are transferability, dependability and confirmability (Parry and Johnson, 2007, Smith, 2016; Wright, 2019). The narrative should also be relevant (topical) and something to which you (the reader) can directly relate (Mienczakowski and Morgan, 2001; Smith, 2016). The evocative language that you are about to encounter has been taken directly from the mouth/mind of an active lifestyle entrepreneur during a nationwide lockdown. The story was co-constructed during an unprecedented period of unrest and uncertainty. It includes extracts from two social media posts shared during and after a couple of COVID-19 lockdowns, the first of which lasted from March to May, 2020. The second lockdown lasted a month, but only applied to people living in the region of Auckland (home to over a quarter of New Zealand's resident population). Passages from the transcript of an in-depth interview conducted prior to the pandemic have been embedded and interwoven into the personal narrative.

The one-on-one interview focused largely on the origins of his lifestyle enterprise and his active entrepreneurial mindset. When answering questions about his background, he was happy to reveal and reflect upon his past experiences and his future aspirations (both professional and personal). It was during that first hour-long conversation where we were able to recognize many of the characteristics and traits associated to active lifestyle entrepreneurship and occupational devotion. Within the interview, he spoke passionately about the company's past, present and prospects for the future. He spoke pleasantly about his employees/colleagues and his clients/customers (with whom he clearly had a lot in common). It was clear that he was doing something that he genuinely loved and that he was loving every minute of what he did. On the subject of work-life balance, he revealed that he had decided to relocate his family out of New Zealand's largest urban center and into one of the nation's most active, yet remote, regional economies. Having worked hard to establish his business, he was looking forward to stepping back a little and spending more time with his family.

The content of the two social media posts add another layer to study that we were in the process of creating from a series of the in-depth interviews. They reveal a side to the individual that wasn't evident during our first meeting. It wasn't hidden (it just wasn't present). The posts were not written by a professional event manager or an experienced business owner-operated trying to impress his audience. They were not written to promote his business venture (or himself). They were, however, full of emotion. They were evocative, provocative and deeply personal. They were raw, real, relevant, most of all, easily relatable. The combined number of positive comments from his colleagues, customers and competitors (n.131), plus the large number of “likes” generated (n.304), tells a story in itself. It told us that he had shared something that other people understood and a story that others had accepted as being both genuine and truthful. It told us that he had written something that other people wanted, perhaps even needed, to see. Collectively, the three narratives allowed us to tell a COVID-19 story yet to be told by our peers within the field of sport events and entrepreneurship. It offered us the chance to illustrate the personal impact of COVID-19 on an active lifestyle entrepreneur who, once upon a time, was looking forward to 2020's arrival for a number of reasons. It was going to be the year his business (referred to as TS) launched two new events and celebrated their 20th anniversary.

Only a small amount of emplotment and editing was applied to the personal narrative that you are about consume. The majority is presented exactly how it was created, including the punctuation, spelling and syntax. For ethical reasons, we have removed all specific references to people and places, including the name of the participant's businesses (referred to as TS). Our narrative was sent back to our entrepreneur on several occasions for feedback and as a form of triangulation. We provided him with plenty of opportunities to critically assess the authenticity and accuracy of our interpretations of his lived experience. A second/follow up interview was deemed unnecessary once we discovered how much quality material he had already provided. Arguably, what makes his personal narrative so powerful and provocative is the fact that it was produced in the moment itself (Smith, 2016). It reveals the fear, anguish and optimism that he was feeling at a time when no one had any real idea how long New Zealand's C-19AL4 lockdown was going to last or, crucially, whether or not it was going to work. If we were to interview him again today, and ask him to reflect upon how he felt during the first lockdown, his answer would inevitably be influenced by everything that has happened since.

### THE Event of Our Lifetime (Got to Run)

Today (the day before my 48th birthday) I am grateful for quite a few things. My body, at the moment it's in OK shape. I'm fit enough to run for 1.5–2 h pretty comfortably. On today's run I thought a lot, I thought about what the fuck has been going on the past 2 weeks. I run trails for a lot of reasons, and one of those reasons is to make sense of the “things” that are going on in my world. I was able to process a lot (well, some) of what this past 2 weeks has done, been, and what it all means.

I really didn't like running when I was at school, so it's kind of bizarre that I started forming this company. I remember it [the start] pretty vividly. Once I made the decision, I went into my bosses office and I suggested that he make my position redundant. I was really fortunate to get that job [as an event manager for the council]. I didn't deserve to get it. I had no experience. But the CEO was a family friend and he decided to take a bit of a shot with me.

I spent two and a half years delivering a portfolio of events, all connected to sport, but not the kind of events that TS does. I went and pitched to him that I would take a number of the events that I knew they wanted to retain, but they didn't really want to organize themselves. I said, “You make my position redundant, I'll organize these events for you, and you can save some money”. I left that meeting with a contract worth twenty grand, which seemed like quite a lot, even though it was a big reduction in my salary.

At that point, I didn't really know how TS was going to look or what we were going to do. I guess for those first few years I kind of just messed around, just trying to make ends up. I just kind of chucked a few things together. I did no planning around preparing a business case or anything mapping out how it might look. If there was a book to be written, it could be around how not to start a business. There wasn't a whole lot of product testing; it was basically just very informal, asking friends “How do you think this could work?” I only just hung in there. I had no responsibilities so I didn't have to pay myself a salary.

I created a concept, and secured the local council as a major funder. That was the start of TS. In the first year, and we're going back 20 years, we got something like 550 people. From there, the portfolio just started to grow and the “Off-road Series” was born with two or three events. I made the decision to stop playing cricket and then replaced that with a little bit of running, a little bit of mountain biking, a little bit more of the endurance kind of stuff. I was always drawn to the off-road stuff or the trail stuff, as opposed to the pounding the pavement kind of stuff. I thought; “Hey, this stuff is kind of fun. Is there an opportunity and would other people kind of buy into this?” A lot of conversations happened with mates over a beer.

I didn't have much support back in those days. All of the events, I've pretty much created by myself in some form. I would map the courses and do the websites and organize the registration; kind of all of it. I would drag people in. It took a couple of years before I had any staff. I'm amazed that some of these events even happened. A lot of it was about instinct, trusting my gut and going, “Hey this is cool, this is a good spot. The people are out there running and they like this kind of stuff; then surely they're going to like this?” I think the key challenge has probably been that a lot of this business has been created and grown pretty organically. I'm always researching what is happening overseas, what's trending.

The biggest challenge for me has been around trying to map out the future of the business; trying to stay ahead of the game a little bit. We were pretty much market leaders for a while, but it's probably fair to say that we've been caught over the years. We were the main people out there offering these sorts of events, but now, oh man, look at all the stuff that's going on there. So, keeping it fresh and innovative has been a massive challenge. We're now a business of ten employees across two offices. Back then the market was pretty uncluttered so we got dibs on putting events on in places before most people.

I think in many ways we popularized trail running in New Zealand. If you were running trails back in those early days, you were kind of a weirdo orienteering guy or girl, or a multi-sporter. Those are small markets and people didn't really get that; so, we were more trying to kind of create something that was a bit more mainstream but still really cool. There was an element of people that were doing it, but they were pretty hardy and they were pretty experienced. Back in those days, you didn't get mothers of three running trails. To create a sanitized safe environment for people to go and do these things was probably the real recipe for success I think.

People that do our events generally have a pretty good time. So, we try to promote the TS part of the business too, as opposed to the events. That's a kind of a romantic view; the view is people will actually potentially make their decision to do an event because they've had a good experience with TS, or one of their friends has and has told them. We do have some very adoring fans, I have no doubt about that, but it's a relatively small percentage. What's actually happens in reality, is loyalty is waning and people identify with the event and if it works for them then they'll do it. So, they will make their decisions based around the event concept, the date and the location and if it fits in with their very busy and full life then they will do it. So, for us, it's constantly about trying to present these events to people. Every event's got a story.

The favorite thing about the business is the people; and the most challenging thing about the business is the people. My role now is to kind of map the future of the business and steer it in the direction it's going to keep it going and hopefully keep it going strongly; which involves being across what's next. We can't continue to run the same events all the time, and if we are then why are we doing what we're doing, how are we keeping them fresh and innovating all the time; so that people actually still consider spending their time and money with us, as opposed to the competition—and there's a lot of that now. There's not a lot of originality left. We are all out there stealing each other's ideas, right? We are continually inspired by the people who do our events and we get their stories from time to time, around what they've been through to get to the start lines and the adversity and the challenge that they've faced.

We like to think that we create inspiring locations and experiences for people. It's about the accessibility too and making it easy. You get a lot of insight around how much people hate their jobs in doing what we do, because they come to us to remove themselves from that stuff that they go through. You hear the stories. I've had a lot of “successful” people come to me who would happily slice their income in half to come and work for something, somewhere like TS. Why? Because they believe in what we do and what we're about. There's this real essence around the people that work here. …you walk upstairs and everyone's kind of got this thing; they all believe and they all know this is a hard business to make money in. It's not a well-paid industry. I'm pretty upfront with these guys that this is not an easy business and we are not smashing it. We're doing okay, but it's a fine line.

I think most of us would agree that 2020 in many ways has been the craziest, hardest and saddest of years. Of course out of adversity comes opportunity and potential, however, all in all it's been tough and it's OK to acknowledge that. I've struggled, my work-mates have struggled, and I've spoken with a lot of people over the last few months that are exhausted, anxious, and just completely over it. I'm not sure about others, but when I start the downward spiral (that's a whole different story!), I let slip a bunch of things that I know 100% are good for me, for both my mental and physical condition. One of those things for me is running, but not just running anywhere—for me to get the full benefits package, I need to get into nature and hit some trails.

I have not been good company lately, not good company at all. Fortunately my wife is “personally developed” to a point where she can let my solemn grumpiness go, she knows it's not about her, and she gets that when things aren't going well, that I become reclusive, non-communicative and extremely dis-engaged, which does not make me a very good person to be around. Luckily our house is pretty big with lots of space and a huge reserve out the front. This means we've got access to space, and that's very, very important right now. My mountain bike has been recently serviced and it's going to get plenty of use over the next month, or more… I'm also very lucky to have a good stock of shoes, I think (hope) I'm going to get through some. I'm sure they're designed to last less time than the olden days. I'm sure the pair I discovered today, that have two big rips in them, have only been in the game for 3–4 months.

Trails are like, so damn important to me, so important. They have been a critical part of my life for the last 20+ years, since TS began. Those trails became the place where we were able to meet, to connect and challenge ourselves in nature, together. So what about my head? Bloody hell. My head has been in some places these last 2 weeks (mostly up my own ass!). I thought I'd try and capture my mental journey to date (and it has felt very MENTAL alright!). Below is my own alert level system 1–4, explained as it has affected and occurred to me and my company.

### Alert Level 1 – Is this Really Happening?

This phase started on Sunday 14th March, 6-days out from our biggest event of the year. The phone started ringing that morning, and it didn't really stop. In my heart I knew that we were very likely going to have to cancel. Those of you that know this event will understand the complexity and challenging nature of delivering an event on the islands. There's something about that wee stretch of water between the mainland and these islands which creates that challenge to produce…! An event for 2000+ outdoor and nature-loving runners, walkers, mountain-bikers and triathletes.

### Alert Level 2 –How the F% and K Do We Deal With This? This Wasn't Meant to Happen to LIL' Old NZ – We're All Good Down This End of the World… Aren't We?

There's a few parts to this phase…

Part one; This bit started on Monday 15th March, when the TS team decided we had no possible way of being able to deliver the event. We made the call to cancel. Well, a week out from an event of this nature, a LOT has been done, and a LOT of $ has been spent (around 70% of the revenue we discovered). We were in the shit. What were we going to do (a) to communicate this cancellation message effectively and authentically to the participants, our sponsors, stakeholders, suppliers, volunteers etc., and (b) to not become bankrupt in the process of canceling this event, the biggest in every sense, in our schedule of 20 events?!

Part two (a); This one started later that same day, when we went into fight-or-flight mode, and scrambled like an egg to work out what our exact, present financial position was. Were we going to be able to claw any $ back from any of our suppliers, and what were we going to offer the many, many disappointed people who'd trusted us, spent their hard-earned money with us, and expected a bloody good show on Sat?

Part two (b); This one started somewhere around Tue 17th or Wed 18th March when it became clear that we weren't going to be putting on our next event, and then very likely the next one after that. The penny started to drop that being in the business of putting on events was perhaps not one of the best businesses to be in right now! During this time my mind went from the micro level (What about that event?, and that event?, and… it's okay… the TS team can deal with that, they're good, like real good!) to the macro level (What does this Covid-19 thing mean for TS?). Very quickly we assessed the financial situation. We came up with our action plan and it was real simple; get every available $ into our bank account that we can, and stop spending any unnecessary $ - immediately. Back onto the phone I went, pleading our case to both our landlords, insurers, and anyone else that we felt we might be able to get some financial respite from. This bit was 100% about survival.

Somewhere in this phase the economic support package was announced and outlined by the government. We were able to tick the wage subsidy bit. There was going to be no problem showing a minimum 30% reduction in income. This is our good time of the year. Financially, times are good, and we're generating an average of $40k+ per week in entry revenue. Since Monday 16th March, we've generated $0. This isn't going to change for a while, so we went from bingo to bugger in a day.

We're not budgeting any revenue for the next 3 months, which is almost exactly how long this business will survive if nothing changes. For now though, we have 12 staff/team members who are working from home (we're not sure on exactly what) and are on 80% of their salary—that's something we can be proud of. We submitted our application on the day that the wage subsidy grants were open (along with thousands of other affected businesses), I got a call within 24-h to check the authenticity of our application, and then the one-off lump sum appeared in our account less than a day later. Less than two days and we at least had a tincy, wincy bit of breathing space.

### Alert Level 3 –I am Feeling Very, Very Sorry for Myself, My World Is in Ruins – Why Isn't Everyone Feeling Sorry for Me?

I'm not exactly sure when this phase started, in all honesty maybe it was on day one (right back at the start). I was just too caught up in trying to deliver positive messages to the team and our community, along with finding a way to keep this company going, to feel it then. But then it kicked in. Once we'd dealt with the messaging, and had an idea of how our next 2-3 months might look, as well as getting just enough $ into our bank account to pay the team, along with a few unavoidable bills, I then got right into the phase of feeling fucking sorry for myself.

I would categorize myself in my “normal” state as an empathetic and a very positive person. Over the past 20 years in business (this business) we'd got through some pretty flippin big stuff. I'd definitely learnt something about resilience. But this time I just jumped straight into a pretty deep pit of self-pity, and I've been here for a while. Why the f%&k did this have to happen?, to the world yeah sure, but to me?, and my company? – this company that does so much good for people, and is turning 20-years old in August! This was the year for celebration, not survival! I've paid my dues, blah blah blah… And all that stuff…

I don't know, some of you might've been here (and maybe still are here) too? And you know what dawned on me somewhere between a hazy IPA and a glass of Shiraz last night?… it's Okay… it's normal to feel this way, and we should give ourselves permission to feel this way. But we can't stay forever in this place of helplessness and deep sympathy for ourselves, because in this place there is zero chance of (a) survival, and (b) future success, once the storm passes. And it will pass.

### Alert Level 4 – Acceptance and Empathy, We Will Get Through this, and Out the Other Side Good Things Will Come

The last phase of my small business owners alert level system started nearly 2 weeks after the shit hit the fan. I woke up feeling good, not just “oh god, can this day just get done so I can go back to bed, to sleep and just escape again for a bit.”1 The feelings of depression and self-pity lifted somewhat, and I felt a whole lot lighter. I felt more present with my wife and my kids. I got to a point where I stopped trying to control this thing, which again can be pretty hard for a person of my nature. People like me like to think we can make things happen when and where the way we want to see them happen. Well, this Covid-19 thing is going to do its thing, and we aren't going to make it go away by telling ourselves that we usually get what we want in this life.

We need to find a way to step back, breathe, do what we can do and roll with these regular punches being dished out. And on the other side… well… I for one really do believe that we'll get back to work at some stage. Someone told me that they thought that we were “in THE event of our lifetimes”, and I believe that. Business, but not just business, all things will be different when C-19 isn't such a thing anymore. Out of adversity and challenge can come opportunity and success and many other good things. I believe that. We'll get back into doing what we love the most, which is getting like-minded people into nature, connecting and challenging themselves together.

Like the rest of the nation (and the world), my time and energy went elsewhere as we attempted to navigate Covid, and it's many, far-reaching impacts. I spent my time trying to fix stuff, and forgot about myself. I don't know much, but I know a few things… especially in times of high anxiety and stress, we NEED this shit, we MUST take the time to stop, to get out into nature and do our thing, whatever that thing might be. I also love mountain-biking and golf, but without a doubt I get the most from running trails. It's so, so good for me.

I enjoy being by myself (I'm a classic introvert), and trails are the place I go to get my solitude fix, to just be me on the trails, with no agenda except to run. I take my issues onto the trail, and it's rare that they come back with me. I can also feel my mind de-fragging while I'm out there, bits get filed into the places where they should be, and create space for me to think more effectively when I head back into the world. At the end of the day, for me it's very basic–I'm a better person when I run regularly. My world becomes balanced and my existence becomes simplified, and I like that!

## Episode Four: A Labor of Love

“The more frequently CAP ethnographies are published, presented, performed, or installed, the more legitimate they become… CAP ethnographies may indeed be the most valid and desirable representation, for they invite people in and open spaces for thinking about the social that elude us now” (Richardson, 1999, p.661).

Atkinson (2002) refers to the healing nature of sharing personal stories as a form of narrative therapy. The principles of Creative Analytical Practice (CAP) are rooted firmly within an interpretivist paradigm built upon the belief that stories play a fundamental role in shaping the personal and the social (Berbary, 2015; Smith, 2016). Critics highlight the lack of generalisability and the lack of familiarity with the phenomenon/phenomena under investigation (Allen-Collinson, 2016). Allen-Collinson, 2016 challenges these criticisms, noting the manner in which personal narratives can fully capture the richness, vitality, evocativeness and grounded “bodyfulness” of the participant's story in a way like no other form of qualitative research. The storyteller, as opposed to the storyanalyst, offers an alternative means of extracting the deeper meanings located within all personal narratives (Smith, 2016).

To conclude, CAP ethnography is a post-qualitative methodology for interpretivists and phenomenologists who accept that no individual method, theory, discourse, genre or tradition can ever be presented as being the best way of capturing the lived experience (Richardson and St. Pierre, 2005). Whilst Laurel Richardson is widely credited as being the mother of CAP ethnography, (Connelly and Clandinin, 1990, p.5) were one of the earliest advocates and adopters of the storytelling approach to narrative analysis, suggesting that “the two narratives of participant and researcher become, in part, a shared narrative construction and reconstruction through the inquiry'. Rather than produce an abstract interpretation of the meanings found within our personal narrative, we propose that the story is both analytical and theoretical in its own right (Smith, 2016; Wright, 2019). As a result, we have chosen to take a back seat and let the voice of our entrepreneurial occupational devotee be the first and focal point of the narrative analysis process (Smith, 2016). The more we signpost our own “answers/conclusions”, and/or justify the method adopted to share this story, the more we risk taking your (the readers) focus away from our active lifestyle entrepreneurs personal experience of COVID-19 (which is the primary focus of the article).

According to Willig (Willig, 2001, p.53), “phenomenological contemplation” focuses on an object or event observed/encountered by the researcher whereas “phenomenological analysis” is solely reliant upon research participant's description of a past lived experience. Whilst the former requires an element of introspection, the latter requires the researcher to temporarily get inside someone else's experience (Willig, 2007). CAP effectively allows the researcher to meet both of these requirements at the same time. This final episode focuses upon the lessons learned and the meaning(s) that we have extracted from what has become a true labor of love. The occupational devotee identifies and immerses themselves within the things they desire and the things that they do to make their existence attractive (worth living) (Stebbins, 2009). In other words, devotee work is a form of serious leisure “from which a full or partial livelihood is possible” (Stebbins, 2014a, p.4).

It is during times of fear and failure that individuals are often forced to temporarily down tools and take stock of their past experiences and present-day priorities. Many people use times of crisis and unexpected change to mentally revisit and emotionally reflect upon where they've been, where they currently find themselves and, perhaps more importantly, where they're heading (Gardner, 2009; Rodham et al., 2020). We have employed CAP in an attempt to inspire other (i.e., you, the reader) to establish your own emotional connection with the evocative content provided (Berbary, 2015; Wright, 2019). Ultimately, we choose to focus on one person's lived experience and utilize a couple of short posts that were also shared via social media. Our findings were never going to be generalisable because that was never our intention. We never started out on this journey with the intention of having to explain/justify or argue the merits and messiness of employing CAP. Our goal was simply to share the story of an active lifestyle entrepreneur caught up in a COVID-19 lockdown. The more we engaged with the serious leisure enthusiast and the method of CAP, however, the more we began to truly love what we were doing. The boundaries between work and play became blurred and we became devoted to sharing this story.

The story that we have shared is expected to resonate with those who found themselves engaging in serious leisure pursuits during a COVID-19 lockdown. We expect it to resonate with small business owners who found themselves feeling lost, angry, alone and/or a little confused by the speed in which their professional world changed (possibly forever). It should strike a chord with those who found themselves having to navigate the practicalities of working from home, whilst also babysitting and/or home schooling their equally lost, angry, frustrated, scared and/or confused children. Finally, we expect that our story will resonate with those who, unlike our active lifestyle entrepreneur, were unable to engage in any serious leisure activities. Moreover, we hope to have targeted your sociological imagination through a personal narrative that illustrates the impact of unexpectedly finding oneself in a COVID-19 lockdown. We hope to have captured your interest in a subject area that we believe deserves more attention within the sport, leisure and tourism academy. We hope to leave you thinking about more than just how the lockdown affected the individual in our story. We hope that you find yourself thinking about your life in lockdown and how it affected your own levels of occupational devotion.

COVID-19 has provided us all with an opportunity to assess our many socially constructed identities, including those related to our professional roles and responsibilities. Although the full impact of the community lockdowns will not be known, or fully understood, for many years to come, we are all in a position to able to critically assess the immediate impact of the social distancing that was forced upon us in 2020. New Zealand's response was, when compared to other nations, highly effective. The number of people who died in New Zealand from COVID-19 in 2020 was recorded by the Ministry of Health as being twenty-five (25) (Ministry of Health, 2021). The focus on stopping the spread, however, came at a significant socio-economic cost. Supply chains were broken. Thousands of jobs were lost. Lives and livelihoods were ruined. Non-essential businesses of all sizes were denied the ability to operate for several months and, once open, they were then forced to operate to a restricted capacity. Even after the lockdowns, the closed borders resulted in businesses only being able to target local customers and domestic tourists.

The New Zealand sport, events, tourism/travel and hospitality sectors were amongst the worst hit, despite the financial support packages provided by the government. Award-winning businesses were left searching for innovative ways of generating new revenue streams. Many were forced to target the local community and domestic tourism market for the very first time. Many more were forced to assess the extent to which they are rooted, if not devoted, to their pre-COVID-19 professional identity. In terms of recommendations, our only one relates to the lack of post-qualitative literature looking at occupational devotion within the sport, leisure and tourism sectors. This would appear to be a missed opportunity, considering the large number of owner-operated (family-run) enterprises found within the service sector, not to mention the large number of lifestyle entrepreneurs choosing to blur the boundaries between work and play (Wright and Wiersma, 2019; Jones et al., 2020). With this in mind, we would like to see more occupational devotion-inspired research being undertaken within the fields of sport, leisure and tourism, including the creation of personal narratives and CAP ethnographies (e.g., autoethnographies and ethnodramas).

## Data Availability Statement

The datasets presented in this article are not readily available because the authors are not permitted to share the recordings of the interview conducted with the participant or the transcripts produced. Requests to access the datasets should be directed to richard.wright@aut.ac.nz.

## Ethics Statement

The studies involving human participants were reviewed and approved by AUT Ethics Committee. The patients/participants provided their written informed consent to participate in this study.

## Author Contributions

RW has overseen the production of this manuscript, which was inspired by the initial findings of a case study and interview conducted by CW. RW has written the majority of it (80%), but RA was brought in to help strengthen Episodes 2 and 4, following the questions and criticisms of one of the reviewers. He has contributed (20%) to the manuscript. Without CW, however, there would be no story. it was her initial engagement with the participant that gave us access and, ultimately, the authority to tell his story.

## Conflict of Interest

The authors declare that the research was conducted in the absence of any commercial or financial relationships that could be construed as a potential conflict of interest.
